# Quantifying accessibility and use of improved sanitation: towards a comprehensive indicator of the need for sanitation interventions

**DOI:** 10.1038/srep30299

**Published:** 2016-07-25

**Authors:** M. J. Park, A. C. A. Clements, D. J. Gray, R. Sadler, B. Laksono, D. E. Stewart

**Affiliations:** 1 Department of Nursing, College of Nursing, Konyang University, Daejeon, South Korea; 2 Menzies Health Institute of Queensland and School of Medicine, Griffith University, Brisbane, Australia; 3 Research School of Population Health, the Australian National University, Canberra, Australia; 4 Yayasan Wahanna Bakti Sehatera (YWBS) Foundation, Semarang, Indonesia

## Abstract

To prevent diseases associated with inadequate sanitation and poor hygiene, people needing latrines and behavioural interventions must be identified. We compared two indicators that could be used to identify those people. Indicator 1 of household latrine coverage was a simple Yes/No response to the question “Does your household have a latrine?” Indicator 2 was more comprehensive, combining questions about defecation behaviour with observations of latrine conditions. Using a standardized procedure and questionnaire, trained research assistants collected data from 6,599 residents of 16 rural villages in Indonesia. Indicator 1 identified 30.3% as not having a household latrine, while Indicator 2 identified 56.0% as using unimproved sanitation. Indicator 2 thus identified an additional 1,710 people who were missed by Indicator 1. Those 1,710 people were of lower socioeconomic status (p < 0.001), and a smaller percentage practiced appropriate hand-washing (p < 0.02). These results show how a good indicator of need for sanitation and hygiene interventions can combine evidences of both access and use, from self-reports and objective observation. Such an indicator can inform decisions about sanitation-related interventions and about scaling deworming programmes up or down. Further, a comprehensive and locally relevant indicator allows improved targeting to those most in need of a hygiene-behaviour intervention.

The final report on the Millennium Development Goals (MDGs) was released in July of 2015^1^. Regarding improved sanitation “the world has missed the MDG target”^1^ with about one-third of the world’s population not using improved sanitation^1^. That is, approximately 2.4 billion people do not use facilities that “are likely to ensure hygienic separation of human excreta from human contact”^2^. This is important for reasons that are well known – a lack of improved sanitation is associated with parasitic, bacterial, and viral diseases. Such diseases can be prevented through programmes to provide, for example, appropriate latrines and education regarding water, sanitation, and hygiene (WASH)^4^,^5^,^6^,^7^,^8^,^9^. One essential task is to assess the need for these programmes, and to evaluate their achievements.

However, assessing relevant needs and achievements is challenging. A simple, straightforward method is to ask people whether they have a household latrine. That approach, however, is likely to be inadequate, for at least two reasons.

Firstly, there is a problem of measurement accuracy. Specifically, the results of such surveys can be confounded by response biases, particularly social-desirability bias, because defecation can be a sensitive topic and also because sanitation reflects socio-economic status^10^. People may report that they have, and use, improved sanitation facilities when in fact they do not^11^,^12^,^13^. Such bias will result in latrine access and use being overestimated, with a corresponding under-estimation of the need for interventions.

Secondly, even accurate information about the presence or absence of a latrine may not be useful. Even if the presence of a latrine is confirmed by a survey worker, the results obtained may not be informative, because health outcomes are not improved by access alone. Behaviour is also important, and people who have access to latrines do not necessarily use them^11^,^12^,^13^,^14^. In addition, the World Health Organization (WHO) clearly recognizes that nominal access to a latrine, such as being allowed to use a neighbour’s or relative’s latrine or use of a public latrine, does not constitute “improved sanitation”^15^.

Such problems undermine the utility of latrine coverage as an indicator of the need for, or the achievements of, programmes to treat or prevent sanitation-related disease. As one step toward overcoming these problems, here we give an example of how self-reports of behaviour can be combined with observation of latrine conditions to yield a comprehensive indicator of the need for therapeutic and preventive interventions. The objective of this study was to compare two possible indicators of that need (not to evaluate interventions). We compared a simple indicator of latrine coverage with a more comprehensive indicator of the use of improved sanitation.

## Methods

We used data collected in a cross-sectional survey of residents of 16 rural villages in Central Java, Indonesia. The survey was conducted to obtain baseline measurements for a separate study (ACTRN12613000523707, a cluster-randomized trial that is assessing the impact of a latrine intervention on infection with soil-transmitted helminths (STH), registered on 10 May, 2013^16^,^17^). A technical description of the latrine used in that trial is in the Appendix. For the present study we analysed that trial’s cross-sectional baseline data from participants aged 13 and over. Socio-demographic information about the participants is presented in Table 1.

Data were collected by 23 research assistants (RAs), who were nurses, midwives, and public-health workers. They underwent 28 hours of training, which included practicing interviews using a standard questionnaire and assessing the physical conditions of household latrines and of home construction in terms of concrete/tile (dry) or earth (wet) floors. Some of the RAs were from the areas being studied and not only shared information about living conditions and daily life in the villages, but also were fully acceptable to village householders and spoke the local language, Javanese, as well as Bahasa Indonesia. When they visited the participants’ homes, the RAs worked in two-person teams, with each member of the team verifying the other member’s observations, and both members checking all recorded data to ensure accuracy. During those home visits, the RAs asked the participants basic socio-demographic questions, questions about their bowel-motion habits, and questions about their hand-washing habits, as hand-washing is particularly relevant to prevention of STH infection^6^. They also asked the participants if they had a household latrine. The RAs noted the physical conditions of latrines used by the participants, if any, and the approximate percentages of dry and wet floor spaces.

To obtain a simple and straightforward indicator of household latrine coverage (Indicator 1), we tabulated the numbers of “yes” and “no” responses to the question “Does your household have a latrine?” So that Indicator 1 might reflect need for sanitation-improvement interventions, it was considered to be positive for participants who responded “no” and negative for those who responded “yes”.

In contrast, to develop a comprehensive indicator of the need for improved sanitation (Indicator 2), we consulted with the village heads and the neighbourhood leaders (*pak-RT*s), and also with the RAs who were responsible for collecting the data. With them, we discussed the villagers’ attitudes toward sanitation and household latrines, and also their actual practices with regard to open defecation, use of shared facilities, and traditional and cultural issues relating to human waste. The discussions took place after the data had been collected. From these discussions we compiled the following list of 6 criteria that reflected either a lack of access to improved sanitation or inappropriate sanitation-related behaviour, or both:

1. having bowel motions in waterways or bush;
2. having bowel motions in neighbours’ or relatives’ latrine (such a “shared” latrine fits the WHO definition of unimproved sanitation)^2^
3. having bowel motions in an unimproved latrine, or a latrine without a septic tank (“*cemplung*”, which can also be translated into English as a “cesspool”. In some cases it may be a shallow hole from which faeces are flushed out by rain, or a latrine from which waste goes directly into surface water bodies.);
4. having bowel motions in a public latrine (which also fits the WHO definition of unimproved sanitation);
5. having a broken home latrine; and
6. having bowel motions in a latrine over a pond or river (the so-called “hanging latrine”^18^).

Answering “No” to the question “Does your household have a latrine” was not included among the criteria for Indicator 2. Participants who met one of the 6 criteria above were considered to be positive on Indicator 2, and those who met none of the 6 criteria were considered to be negative on Indicator 2.

We compared Indicator 1 with Indicator 2, and also compared both indicators with the results for rural Indonesia that were reported most recently by the WHO^2^. If a participant responded “yes” to the question about having a household latrine (i.e., negative on Indicator 1) but still met one of the 6 criteria of not using improved sanitation (i.e., positive on Indicator 2) then the results on the two indicators were considered to be discrepant. In this study, the main analyses are focused on the participants whose results were discrepant. A multiple logistic regression model was constructed, with the dichotomous outcome being “discrepant vs negative on both indicators”, and with the potential predictors being socio-demographic variables and responses to the questions about hand-washing practices. Hand-washing practices were included in the model because they are directly relevant to prevention of STH infection^6^.

Data were analysed using IBM SPSS Version 22 and Microsoft Excel.

This study was carried out in accordance with relevant guidelines, and informed consent was obtained from all participants. Ethics approval was obtained from Griffith University’s Human Research Ethics Committee (PBH/17/11/HREC) and from Diponegoro University (068/EC/FK- RSDK/2014).

## Results

Although the response to the question about having a household latrine was not included among the criteria for Indicator 2, all of the participants who answered “no” to that question did meet at least one of the 6 criteria for Indicator 2.

As shown in Table 2, Indicator 1 identified 1,984/6,599 people (30.1%) as not having a household latrine. In contrast, Indicator 2 identified 3,694 people (56.0%) who were not using improved sanitation. Two components of Indicator 2 were defecation in “waterways & bush” (1,376 people) and *cemplung* (841 people), both of which meet the WHO definition of open defecation. Thus, an estimated total of 2,217 people were practicing open defecation, which is 33.6% of the total.

No hanging latrines were observed by the RAs, and none of the participants reported using one.

The indicators were discrepant in 1,710 participants (25.9%). About half of them reported that they had bowel motions using a neighbour’s or relative’s latrine, and about half used a *cemplung*.

In Table 3, the participants who answered “Yes” to the question about having a household latrine and met none of the criteria of Indicator 2 (i.e., those who were negative on both indicators) are compared with those for whom the two indicators were discrepant. Participants in the latter group were more likely to have lower levels of schooling (p < 0.001), to have lower household income (p < 0.001), and to be living in a home with less than 25% dry floor space (p < 0.001). They were also less likely to report practicing appropriate hand-washing (p < 0.02 for all 6 questions about hand-washing).

Table 4 shows the logistic regression model. Among the predictors of being discrepant on the two indicators were household income, formal education, and the use of soap in hand-washing.

## Discussion

A simple indicator of household latrine coverage among 6,599 residents of 16 rural villages in Central Java identified a substantially smaller number of people than a comprehensive indicator of the use of unimproved sanitation.

An important characteristic of the comprehensive indicator is that it reflects both self-reported behaviour and objectively observed physical aspects of residents’ sanitation facilities and living conditions. Collecting additional information for the comprehensive indicator required extra investment of time and effort. However, one quarter of the participants in this survey (1,710/6,599 = 0.26) had a need to use improved sanitation that was not reflected in the indicator of household latrine coverage, but was recognized with the comprehensive indicator.

Of the comprehensive indicator’s 6 criteria, one was not met by any of the participants: use of a hanging latrine. Whilst hanging latrines were not found in this area of Indonesia, they are relatively common in some parts of India and Bangladesh and they have been observed in other parts of Indonesia^19^,^20^. The area in which this study was done has many hills, where the streams tend to be fairly small and not amenable to hanging latrines, unlike flat land and coastal areas. To ensure broad applicability of Indicator 2 within Indonesia, we would not remove hanging latrines from the list of criteria for unimproved sanitation.

Unlike the result from Indicator 1 (30.3%), the result from Indicator 2 (56.0%) was close to the 54% reported by the WHO to be using unimproved sanitation in rural Indonesia as a whole^2^. Additionally, the percentage practicing open defecation as identified by Indicator 2 (33.6%) was close to the percentage reported for all of rural Indonesia (31%)^2^.

### Direct and indirect indicators

Optimally, the best method to assess needs might be to assess directly, using laboratory methods, the presence or absence of water-borne and soil- borne pathogens. Unfortunately, outside of well-funded research projects the time and cost involved can make such direct assessments impractical for large populations. Alternatively, we can investigate indirect measures. Specifically, because it is a variable that may be only one step removed from health outcomes, not using improved sanitation can be seen as an indirect indicator of a need for interventions against water-borne and soil-borne pathogens. The WHO definition of improved sanitation certainly provides a reasonable general definition but we suggest that it must be interpreted in every local context. The 6 criteria used here to comprise Indicator 2 can be seen as one example of a specific, operational definition for detecting the use of unimproved sanitation.

### Participants for whom the two indicators were discrepant

One practical consequence of using a comprehensive rather than a simple indicator can be seen from examination of the people in whom the two indicators were discrepant, that is, they responded “Yes” to the question about having a household latrine, but were not using improved sanitation, according to Indicator 2. Tables 3 and 4 both indicate how these were different from the other participants. Compared with the 2,905 participants who were negative on both indicators, the 1,710 for whom the two indicators were discrepant appeared to be of lower socioeconomic status, with lower household incomes, less formal schooling, and a higher percentage living in homes in which 75% or more of the floor space was beaten earth (Table 3). Thus, using Indicator 1 alone would result in the needs of the most economically disadvantaged residents of these rural villages receiving less attention.

In addition, we note that smaller percentages of the participants for whom the two indicators were discrepant reported that they practiced appropriate hand-washing. This we take as evidence suggesting that using Indicator 1 would also underestimate the need for interventions to improve hygiene-related behaviour. Such underestimation might be even greater than implied by these results, as social-desirability bias could have caused over- reporting of hand-washing.

### Example of a possible application: scaling-down deworming programmes

Comprehensive indicators (e.g., this study’s Indicator 2) can be used to inform public health decisions. One example would be decisions about whether to scale-down programmes of deworming to treat STH infections, which remain an important problem in many areas^21^,^22^,^23^. Such programmes are often considered to be worthwhile, but deworming is quickly followed by re-infection^24^. Scaling-down of deworming programmes is necessary to avoid unnecessary treatment of STH-controlled communities, to reduce donor fatigue, and to minimize risks of drug resistance developing. In this context it is important to have good indicators of when deworming is no longer needed. Using improved sanitation lowers the risk of STH re-infection, so areas in which all or almost all residents are negative on this study’s Indicator 2 would be areas in which existing deworming programmes might be scaled down. Using Indicator 2 rather than latrine coverage (Indicator 1) would result in relatively safe, conservative decisions about scaling down of deworming programmes. Specifically, there will be fewer false negatives, where deworming is incorrectly judged to be no longer needed. This is because, unlike Indicator 1, Indicator 2 has multiple criteria applied in parallel and thus its sensitivity is relatively high^25^. High sensitivity is an important advantage of Indicator **2**, because the low false-negative rate would help to ensure that the programmes are not scaled down prematurely.

Deworming programmes should be followed by programmes to prevent STH re-infection. Such programmes should focus on water, sanitation, and hygiene^26^, and this study’s Indicator^2^ could be useful in evaluating those programmes, because it shows the use of improved sanitation directly.

### Generalisability, local relevance, and research staff

One aspect of good indicators is that they are locally relevant. Good indicators of the use of unimproved sanitation should take into account social norms and local cultural practices regarding defecation. Also important are climate, population density, local geography, and mode of subsistence (rice farming, animal husbandry, occasional labour in peri-urban areas, etc.). However, local relevance inevitably makes indicators less generalisable. For example, it would not be reasonable to expect the rural Indonesian concept of *cemplung* to be useful when assessing sanitation needs in urban slums of sub- Saharan Africa^27^.

To make a single indicator that would be useful worldwide, one would need a list of criteria for detecting the use of unimproved sanitation in any human-ecological context. For such a list to have specific criteria while being universally applicable is probably unrealistic. Despite some overlap between countries (e.g., the use of hanging latrines in different areas), even if such a list were compiled it would likely be too long for practical use.

Even though local relevance of an indicator may be gained only with a loss of generalisability, we believe that the *approach* we used to develop Indicator 2 is generalisable. Specifically, we expect that in many human-ecological contexts locally-relevant indicators can be developed in accord with two main characteristics of this study’s approach: (1) assessing not only access to improved facilities but also behaviour, and (2) using local knowledge to translate general statements (such as the WHO definition) into specific, locally-relevant criteria. As for the latter, the contributions of the RAs to this study should not be underestimated: they not only collected the data but also provided crucial insights that led us to develop the comprehensive indicator. This is, we believe, an important point shown by the present work: the benefits of careful recruiting, staff motivation, thorough training, and in- depth discussions about local culture in context.

## Conclusions

Overall, we interpret the difference between the two indicators as reflecting a gap in utility between the concepts of latrine coverage and of using improved sanitation. It is a gap between, on the one hand, an indicator that is quite easy to obtain and, on the other hand, one that requires more effort to measure but is likely to be more useful. We believe that Indicator 2 allowed a more accurate identification of those needing treatment and sanitation-related interventions. Based on these findings, we would propose, first, that practical and robust indicators should reflect both access and behaviour assessed both from self-reports and by trained observers, and second, that those indicators should be developed collaboratively to incorporate local knowledge.

## Additional Information

**How to cite this article**: Park, M. J. *et al.* Quantifying accessibility and use of improved sanitation: towards a comprehensive indicator of the need for sanitation interventions. *Sci. Rep.* **6**, 30299; doi: 10.1038/srep30299 (2016).

## Supplementary Material

Supplementary Information

## Figures and Tables

**Table 1:** Socio-demographic information (n = 6,599).

Variable	Category or measure	Value
Gender	Male	3,273 (49.6%)
	Female	3,326 (50.4%)
Age (missing n = 23)	Mean ± S.D.	39.2 ± 17.3
	Range	13–93
Schooling (missing = 46)	No schooling	915 (13.9%)
	Elementary school	2,540 (38.5%)
	Junior high school	1,595 (24.2%)
	Senior high school	1,317 (20.0%)
	College or higher	186 (2.8%)
Household income/month*	Median (25–75%)	Rp. 1,000K (600K-1,500k)
		USD 74.8 (44.9–112.3)
Dry floor space of home*	= < 25%	964 (39.9%)
	>25%	1,455 (60.1%)

*Data from each household (n = 2,419).

**Table 2:** Results from applying Indicators 1 and 2.

Indicator 1 (simple): Household latrine coverage			
Criterion	Yes	No	P value
Response to “Does your household have a latrine?”	4,615 (69.9%)	1,984 (30.1%)	
Household income			
Median (25–75%)	1,000K (800K–1,500K)	1,000K (600K–1,200K)	<0.001*
Dry spaces			
= < 25% dry spaces	1,121 (24.3%)	1,399 (70.5%)	<0.001**
>25% dry spaces	3,494 (75.7%)	585 (29.5%)	
Indicator 2 (comprehensive): Not using improved sanitation			
Criterion	Number meeting the criterion	Number discrepant between Indicator 1 and Indicator 2*	
1. Waterways & bush	1,376 (37.5%)	16 (0.9%)	
2. Neighbours’ or relatives’ latrine	1,191 (32.2%)	824 (48.2%)	
3. *Cemplung*	841 (22.6%)	836 (48.9%)	
4. Public latrine	260 (7.0%)	12 (0.7%)	
5. Home latrine is broken	26 (0.7%)	22 (1.3%)	
6. Hanging latrine over pond/river	0	0	
Total	3,694	1,710	

*Mann-Whitney U test (The distributions of household incomes were right-skewed: Kolmogorov-Smirnov test p < 0.001 for both the “Yes” group and the “No” group.); **Chi-square test.

*The number discrepant between the two indicators is the number of people who responded “yes” to the question about having a household latrine, but nonetheless were not using improved sanitation according to Indicator 2.

**Table 3:** Comparison between the people who were discrepant between the two indicators and those for whom the two indicators were the same.

Variable	Positive on both indicators	Discrepant*	Negative on both indicators	Chi-square (df), or Mann-Whitney U (z), **
	(n = 1,984)	(n = 1,710)	(n = 2,905)	*p* value
Gender (n and % who were female)	987 (49.7%)	864 (50.5%)	1,475 (50.8%)	—
Age (mean ± SD; range)	39.6 ± 18.3	40.3 ± 17.8	38.4 ± 16.2	—
	13–93	13–91	13–90	
Schooling	n = 1,969	n = 1,693	n = 2,891	111.8 (4)
None	379 (19.2%)	270 (15.9%)	266 (9.2%)	<0.001
Primary school	798 (40.6%)	695 (41.1%)	1,047 (36.2%)	
Junior high school	485 (24.6%)	411 (24.3%)	699 (24.2%)	
Senior high school	292 (14.8%)	288 (17.0%)	737 (25.5%)	
College, or higher	15 (0.8%)	29 (1.7%)	142 (4.9%)	
Monthly household income (Rp)	1,000K	1,000K	1,200K	1,815,447 (−14.0)
Median (25–75%)	(600K–1,200K)	(600K–1,400K)	(900K–1,650K)	<0.001
Dry floor space = < 25%	1,399 (70.5%)	1,094 (64.0%)	27 (0.9%)	2,326.6 (1)
				<0.001
Always washing hands after bowel motion	597 (30.1%)	627 (36.7%)	1,186 (40.8%)	7.8 (1)
				0.005
Always washing hands on coming back home	633 (31.9%)	651 (38.1%)	1,212 (41.7%)	5.9 (1)
				0.015
Always washing hands before eating	1,007 (52.3%)	1,017 (59.5%)	1,894 (65.2%)	15.1 (1)
				<0.001
Always washing hands after eating	810 (40.8%)	828 (48.4%)	1,541 (53.0%)	9.2 (1)
				0.002
Always washing hands before praying	1,044 (52.6%)	960 (56.1%)	1,753 (60.3%)	7.8 (1)
				0.005
Always using soap	901 (46.1%)	960 (56.1%)	1,873 (64.5%)	31.5 (1)
				<0.001

*“Discrepant” means negative on Indicator 1 (i.e., “yes” to the question about having a household latrine), but positive on Indicator 2 (i.e., met one of the 6 criteria). None of the participants was positive on Indicator 1 and negative on Indicator 2.

**Comparison of the discrepant group with the group that was negative on both indicators. The distributions of monthly household incomes were right-skewed: Kolmogorov-Smirnov test p < 0.001 for both groups. Monthly household incomes were compared with the Mann-Whitney U test. The other variables were analysed with Pearson’s chi-square test.

**Table 4:** Multiple logistic-regression model (dependent variable: discrepant vs. negative for both indicators)

Independent variables	Coefficient (β)	Standard error	Wald χ^2^	*P* value	Adjusted odds ratio (95% confidence interval)
Intercept	−1.34	0.11	—	—	—
Gender	0.69	0.06	1.15	0.28	1.07 (0.96–1.21)
Age	−0.006	0.002	8.33	0.004	0.994 (0.989–0.998)
Schooling	0.28	0.04	57.18	<0.001	1.32 (1.23–1.42)
Household income	0.59	0.07	74.14	<0.001	1.81 (1.58–2.07)
Washing hands after bowel motion	−0.12	0.08	2.14	0.14	0.88 (0.75–1.04)
Washing hands on coming back home	−0.03	0.08	0.10	0.75	0.98 (0.84–1.14)
Washing hands before eating	0.10	0.10	1.02	0.31	1.10 (0.91–1.33)
Washing hands after eating	0.12	0.09	1.77	0.18	1.13 (0.95–1.35)
Washing hands before praying	0.13	0.07	3.01	0.08	1.14 (0.98–1.31)
Using soap	0.27	0.07	14.89	<0.001	1.31 (1.14–1.51)

Coding of dependent variable: negative on both indicators = 0, discrepant = 1.
