# Supplementary material for: Quantifying accessibility and use of improved sanitation: towards a comprehensive indicator of the need for sanitation interventions
Source: Sci Rep. 2016 Jul 25;6:30299. doi: 10.1038/srep30299 (PMC4958982; doi:10.1038/srep30299)
Supplement: Supplementary Information [file srep30299-s1.doc]

Quantifying accessibility and use of improved sanitation: towards a comprehensive indicator of the need for sanitation interventions

M.J. Park^1,2^, A.C.A. Clements^3^, D.J. Gray^3^, R. Sadler^2^, B. Laksono^4^, D.E. Stewart^2^

^1^Department of Nursing, College of Nursing, Konyang University, Daejeon, South Korea

^2^Menzies Health Institute of Queensland and School of Medicine, Griffith University, Brisbane, Australia

^3^Research School of Population Health, the Australian National University, Canberra, Australia

^4^Yayasan Wahanna Bakti Sehatera (YWBS) Foundation, Semarang, Indonesia

**Supplementary information: A cheap, easily made family/household latrine, usable in both wet and dry conditions (the ‘BALatrine’).**

**Overview**

Budi’s Amphibious Latrine (the ‘BALatrine’) is a simple but effective household latrine designed to be made by village residents using local materials in resource-limited developing country villages. It is congruent with the users’ habits, funds and environment. As an ‘amphibious’ model, the BALatrine can be used whether water is available or not. It is inexpensive and can be copied by people with very limited income, or it can represent an excellent, cheap but effective ‘buy’ for government instrumentalities or private agencies.

Previous research in the Central Java village environment in Indonesia identified a number of reasons for the lack of success of many latrine campaigns [1]. These included the lack of flexibility regarding the availability of water to assist human waste disposal in a country or region that experiences both wet and dry seasons; the need for cultural familiarity, simplicity and ease of use; and the need for a latrine to be affordable by all households. The BALatrine also provides ‘improved sanitation’, defined as one that hygienically separates human excreta from human contact. It has been developed and modified over more than a decade to address these issues and to ensure that existing unsanitary and unhygienic (non-latrine) customs and habits are discouraged and that villagers, through ownership and implementation of the BALatrine, adopt new, healthier sanitary toilet habits.

A key feature of the BALatrine is that it is designed to be adopted at the household or family level, and therefore requires the engagement of householders at a number of levels. This includes, for example, their personal ownership of the decision to invest time and money in a household latrine, to install it and to use and maintain it in a hygienic and healthy manner. At the household level, when a family makes the decision and the financial investment to build a family latrine, the owner will typically choose to build it near his house and this ownership and investment will mean better maintenance. Thus when, for example, a child uses the latrine and does not flush it, the whole family accepts responsibility for latrine hygiene and this, in turn, means a more hygienic environment and better health for the whole family. It effectively shifts villagers from a pre-contemplation, or contemplation stage of change, to participatory action and engagement.

The latrine may also be used in natural disaster or refugee camp situations, as took place during and after the 2010 Mt Merapi (Java) volcanic eruptions, because it is quick to construct and just as quick to close and, as indicated above, is independent of water availability [2].

**Design**

Much of the technology and most materials required for construction are available in the village context, such as mattock, crowbar, handsaw and trowel, small pieces of timber or bamboo, cement, sand, split stone and pieces of PVC pipe. Some of the materials may need to be supplied, in part, by a supporting government or agency, however these costs are relatively low (around US$15-20.00). As a typical ‘squat’ latrine, the construction is simple and consists of a septic pit or tank; a concrete plate, or mould; and a removable U-bend (goose-neck) water closet/barrier.

The pit is dug to a depth of two or more metres depending on the fluctuation of local water levels, with the bottom of the pit above the local water table and at least 20 metres from any household or village well (see Figure 1). In soil that is easily eroded, the pit can be strengthened with bamboo or cement (see Figure 2). Over the pit, a moulded plate is placed which offers a ‘dry’ option when there are water restrictions or other issues limiting the use of water to flush the latrine (see Figure 3).

Figure 4 illustrates the latrine plate from two perspectives. If the plate only is used, a lid is required to minimise insect involvement and odours. While this is less hygienic than the added water closet/barrier (see Figure 5), it is a major improvement on polluted gardens, tracks and waterways. As Figure 5 illustrates, the water closet/u-bend barrier is placed on top of the base plate and may be either fixed as permanent, where water supply is regular and assured; or be removable, where dry season or water restrictions are frequent or regular (see Figure 7).

The addition of a water closet/U-bend barrier (see Figure 6) allows the latrine to be used in wet conditions, or when a reasonable water supply is available. This aspect of the model is similar to the usual latrine typically recommended by health officers and is usually made of porcelain. A water reservoir is not recommended as this often raises the problem of mosquitoes in the home environment.

**Construction materials and method**

1. Tools and materials - All tools and materials are easily available in the village environment and are simple and hand-held, see Table 1.
2. Construction
3. Construction of the septic tank (see Figures 1 & 2):

- Make a hole with a diameter of 1m and a depth of 2m or more
- The bottom of the hole should be above the water table in the surrounding land.
- The minimum distance from a well should be 10m.
- If the land is easily eroded, the hole should be strengthened with bamboo or cement.

(ii) Construction of the BALatrine plate (see Figures 3 & 4):

- Cut the bamboo into 115cm lengths; split it into 4 pieces. Then plait it at 20cm intervals. Alternative similar local materials may be used as a substitute.
- Cut the wood board into 120 cm, 4 pieces. Make a square and nail it. This is used as a mould for the plate.
- Place the plaited bamboo into the wooden square. Place small stones under the bamboo to ensure the bamboo will be well covered by the concrete.
- Make concrete by mixing cement, sand and split stone in proportions of 1:2:3. Make sure that the mixture is well combined.
- Make the hole and foot rest moulding in the middle.
- Place the PVC pipe in corner. This is used for ventilation of the biological processes in the septic tank.
- Pour the wet concrete into the wood square. Ensure the concrete covers all of the bamboo. The thickness of the concrete should be 15 cm.
- Fill the foot rest with the concrete.
- Allow to set for 24 hours before removing the moulding. (It is possible to remove moulding 2 hours after pouring concrete if the mould needs to be used again, but be certain to protect the wet concrete from possible damage.)

(iii) Construction of the water closet or goose-neck/u-bend barrier (see Figures 5 & 6):

- Prepare the ‘goose-neck’ mould for the amphibious closet. Use Vaseline or paper to ensure easy removal.
- Put the goose-neck u=-bend into the mould in an inverted position.
- Fill the mould with wet concrete mixture.
- When the mixture reaches about 12 cm from the top, put the foot rest mold in place.
- Continue the filling of concrete until the foot rest connects with the latrine plate.
- When the concrete is dry, remove the mould.

When the concrete is strong enough, take the goose-neck/u-bend barrier carefully and put it above the hole of the septic tank. The positioning of closet should take into consideration the household interior orientation/plan and local habits. Local customs may dictate placement directions. Because of the weight and fragility of the concrete while still wet (full drying period recommended is 28 days), four people or more may be needed to position it. Care must be taken to keep it level on all sides during positioning to prevent damage. Once the plate is in place, position the amphibious goose-neck/u-bend barrier over the latrine’s plate.

**Conclusion**

The BALatrine is a standard squat latrine model, with the difference that it can accommodate the availability or lack of water, together with relatively low maintenance as far as cleaning is concerned. The amphibious closet may be removed when flushing is not feasible and matter is deposited directly into the septic tank hole. As a precaution against flies and other insects, the hole must be secured with a wooden cover. Although not ideal, latrines built according to these specifications are preferable to open sanitation and pollution of natural waterways and, in terms of health promotion, they allow time for villagers to become adjusted to improved health standards, awareness and practice.

Communal use without sufficient cleaning responsibilities degrades the condition of any latrine. The BALatrine, as a household-based facility with personal ownership, avoids the problems of Government-funded latrines towards which individuals feel no ownership or responsibility for cleaning, or to spend money to fix them when broken. There are advantages and disadvantages to the BALatrine, but if individuals or communities are not ready to receive a permanent latrine for whatever reason, including water restrictions, habits, culture, lack of social awareness and enforcement of sanitary planning and regulations, this model offers an effective approach.

**References**

1. Stewart, D. & Laksono, B. (2002) “Helminth infection, human waste and appropriate

technology: An Indonesian case study”. *Environmental Health*, Vol. 2, No. 4, pp. 46-52.

1. Fatoni, Z & Stewart DE (2012) Sanitation in an emergency situation: a case study of

the eruption of Mt Merapi, Indonesia, 2010. *International Journal of Environmental*

*Protection*. Vol. 2 Iss. 6, pp. 1-5.


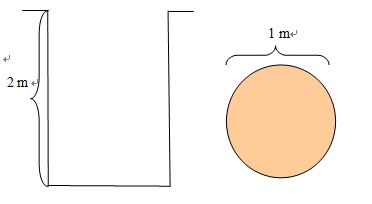


Figure 1: Dimensions of the Septic pit


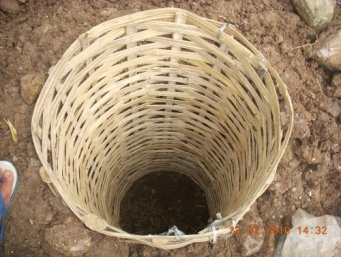


Figure 2: Septic pit with bamboo strengthening

Figure 3: Construction of the moulded plate


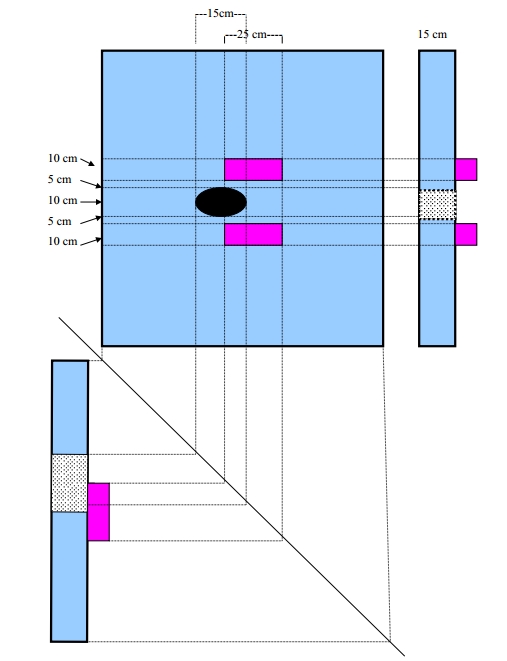


Figure 4: Latrine plate that fits over the Septic pit from 3 perspectives


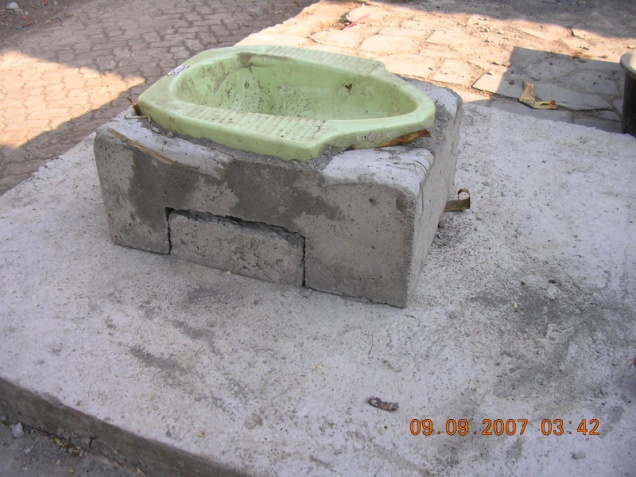


Figure 5: Positioning the water closet on the plate


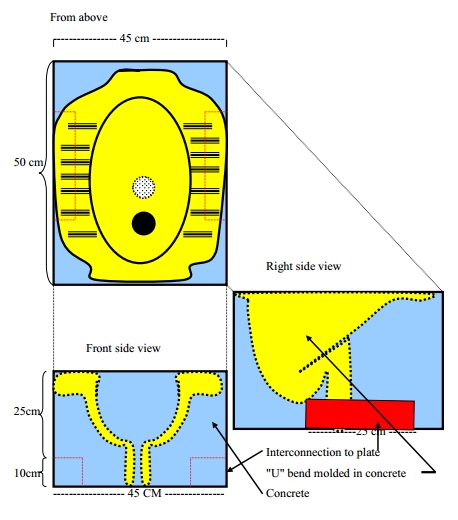


Figure 6: Water closet or ‘amphibious’ goose-neck/u-bend barrier from three perspectives

Figure 7: Flexibility of the wet/dry latrine option

| *Tools* | ·         Mattock |
| --- | --- |
|  | ·         Crowbar |
|  | ·         Wood hand saw |
|  | ·         Trowel |
|  | ·         Budi’s amphibian latrine molding |
| *Materials* | ·         Bamboo poles (½ length) |
|  | ·         Goose-neck porcelain bowl (1 piece) |
|  | ·         Cement (1, 126 sack (40 kg)) |
|  | ·         Sand (0.1348 M3) |
|  | ·         Split stone (0.2248 M3) |
|  | ·         PVC pipe. |

Table 1 Construction tools and materials
